# Echocardiographic Measurements in Normal Healthy Adult Population of North India

**DOI:** 10.7759/cureus.47449

**Published:** 2023-10-22

**Authors:** Aamir Rashid, Aejaz A Shah, Hilal Rather, Vamiq Rasool, Imran Hafeez, Shahood Ajaz, Sameer Purra, Ajaz A Lone

**Affiliations:** 1 Department of Cardiology, Sher-i-Kashmir Institute of Medical Sciences, Srinagar, IND

**Keywords:** kashmiri, healthy, measurements, echocardiographic, normal

## Abstract

Background and aim: Interpretation of imaging modalities depends on robust normal reference limits. Ethnicity is an essential determinant of cardiac chamber sizes. Though few studies from India have focused on this research, it has yet to include the Kashmiri population. We aimed to study normal echocardiographic values of healthy Kashmiri adults and compare them with Western and Indian studies.

Methods: It was a prospective observational study on healthy adults of Kashmir Valley. A comprehensive echocardiographic analysis following standardized protocols was performed.

Results: A total of 2245 study participants were analyzed. The mean age was 32.52±11.55 years. There were 1100 (49%) males. Males had higher absolute left ventricular volumes and mass, left atrial volumes, right ventricular diameter, and aortic size, while females had higher absolute left ventricular ejection fraction and early and late diastolic mitral inflow velocities. Males had higher indexed left ventricular end-systolic volume, while females had higher indexed left ventricular end diastole diameter, aorta diameter, right ventricle, and left and right atrial sizes. Left ventricular mass and diastolic parameters were significantly associated with age. Compared with the American Society of Echocardiography/European Association of Cardiovascular Imaging, absolute values of left ventricle size, volumes, mass, right ventricle size, aortic size, and left and right atrial size were higher than those in our study. Our study population had a higher left ventricle ejection fraction. Among indexed parameters, left ventricle volumes, left ventricle systolic diameter, aortic annulus, and left and right atrial volumes were still significantly higher in Western data. While comparing with Indian data, we noted significant regional differences.

Conclusion: We provide normal reference values for our local population. We noted significant differences with Western as well as other Indian populations. Our study highlights the need for developing ethnic-specific reference values of various echocardiographic measurements.

## Introduction

Medical, interventional, or surgical management decisions are widely based on echocardiography results for cardiac and noncardiac patients. Therefore, to detect abnormalities, an accurate definition of normal values of echocardiographic measurements is of utmost importance to be a reliable guide for decision-making [1-4].

Normal values for echocardiographic measurements have been presented in several guidelines and statements. The most recent was a joint recommendation for chamber quantification by the European Association of Cardiovascular Imaging (EACVI) and the American Society of Echocardiography (ASE) [5]. Differences have been shown to exist in some echocardiographic measurements that could be attributed to racial, ethnic, or gender differences [5-7].

Earlier studies have shown that ethnicity is an essential determinant of cardiac chamber sizes. Indians have been shown to have smaller chamber sizes than Europeans but equivalent or higher left ventricular ejection fraction (LVEF) [8-10]. It was observed earlier that left ventricular end-systolic volume index (LVESVI) and left ventricular end-diastolic volume index (LVEDVI) were smaller in Indian Asian males and females than their European white counterparts, while LVEF was similar between ethnicity-sex subgroups [9]. It was also observed previously that indexing to body surface area (BSA) reduced the left ventricular end-diastolic volume (LVEDV) and left ventricular end-systolic volume (LVESV) differences between Indian measurements and ASE-defined normal considerably [3]. These references make a strong point for collecting different population-based normative data useful for comparison and reference by the medical community.

Though few studies from India have focused on this research, none has included native Kashmiri population [8,10-12]. In this study, we present the normal echocardiographic values of healthy Kashmiri adults and compare them with the Western and other Indian populations to detect any ethnic differences.

## Materials and methods

This prospective study was conducted on normal, healthy adults of Kashmir Valley in our tertiary care institute by the Department of Cardiology from May 2019 to December 2021. The local hospital ethical committee cleared the project. Written informed consent was obtained from all the participants.

Study subjects

The participants included normal, healthy adults from hospital staff, students, attendants accompanying patients, and people who randomly came for a cardiac medical check-up and had no cardiovascular disease history. All such consecutive participants who consented to the study were recruited. A convenient sampling method was used to recruit participants. The workup comprised basic clinical and biochemical parameters. The following parameters were recorded: height, weight, body mass index (BMI), body surface area (BSA), complete blood count, urea, creatinine, creatinine clearance, blood sugar fasting, lipid profile, and electrocardiography (ECG). Initially, 3025 patients were assessed for eligibility. Of these, 225 study subjects declined to participate. A total of 555 subjects were excluded due to various reasons, including hypertension in 250, lung disease in 30, renal dysfunction in 100, diabetes mellitus in 30, known cardiovascular disease in 100, and hypothyroidism in 45 patients. Finally, 2245 study participants were allocated and analyzed.

Exclusion criteria

The subjects were excluded based on the following criteria: (1) subjects who had any cardiovascular disease like congenital, valvular (except trivial regurgitation), cardiomyopathy (dilated, ischemic, hypertrophic, infiltrative or restrictive), ischemic wall motion abnormalities, arrhythmias, diastolic abnormalities, and abnormal systolic LV function; (2) systolic blood pressure ≥140 mmHg, diastolic blood pressure ≥85 mmHg, or history of drug-treated hypertension; (3) diabetes or impaired fasting glucose ≥100 mg/dL; (4) body mass index ≥30 kg/m^2^; (5) creatinine ≥1.3 mg/dL, estimated glomerular filtration rate ≤60 mL/min/1.73 m^2^; and (6) total cholesterol ≥240 mg/dL, low-density lipoprotein cholesterol ≥130 mg/dL, and total triglycerides ≥150 mg/dL.

Two-dimensional TTE

We adopted a standard protocol for performing the two-dimensional trans-thoracic echocardiographic study (2D TTE) studies with machine-integrated ECG recording using Vivid S5 machines with an M3S matrix array probe with a frequency range from 1.7 to 3.2 MHz (GE Vingmed: Horten, Norway). A comprehensive echocardiographic study, conducted according to standardized protocols [5,13], included measurements taken in accordance with ASE guidelines [5] and comprehensive trans-thoracic echocardiography examination guidelines provided by the Indian Academy of Echocardiography [14]. The echocardiography/Doppler examination was conducted in parasternal long- and short-axis views and the three standard apical views. Three consecutive cardiac cycles were recorded during quiet respiration in each view in the left lateral decubitus position. Measurements for the left ventricular posterior wall thickness at end-diastole (PWD), interventricular septum at end-diastole (IVSD), left ventricular internal dimension at end-diastole (LVEDD), and left ventricular internal dimension at end-systole (LVESD) were obtained by two-dimensional method. Measurements were made from the leading edge of the septal endocardium to the leading edge of the posterior wall endocardium [5]. The stroke volume (SV) and cardiac output were measured from the left ventricular outflow tract (LVOT) tract or aortic annulus. SV was derived as follows: SV = cross-sectional area (CSA) × velocity time integral (VTI). From the parasternal long axis view, anteroposterior left atrial diameter (LAD) was measured perpendicular to the aortic root long axis, at the level of the aortic sinuses by using the leading-edge to the leading-edge convention at end-systole, just before mitral valve opening representing the maximal left atrial volume. In addition, aortic root diameter (at the maximal diameter of the sinuses of Valsalva, annulus, and sinotubular junction) was obtained from the same view. Apical four-chamber M-mode echocardiography at lateral tricuspid annulus was used to measure tricuspid annular plane systolic excursion (TAPSE). Mitral E velocity (mitral inflow early diastolic velocity), mitral A velocity (mitral inflow late diastolic velocity), deceleration time (DT), and E/A ratio were obtained using pulsed Doppler spectral recording at the tips of the mitral valve in an apical four-chamber view. Further, e’ (mitral annular early diastolic velocity) and a’ (mitral annular late diastolic velocity) velocities were measured at the medial mitral annulus and lateral annulus using tissue Doppler imaging (TDI). Left atrial end-systolic volume (LAV), left ventricular end-diastolic volume (LVEDV), left ventricular end-systolic volume (LVESV), and LVEF were measured using Simpson’s biplane (two- and four-chamber views) method of discs in end-diastolic and end-systolic frames. Two-dimensional speckle tracking echocardiography (2D-STE) was performed using ECG gating on the three standard apical views (four-chamber, two-chamber, and three-chamber views) using automated cardiac motion quantification to measure global longitudinal strain (GLS). Right ventricle (RV) dimensions (including right ventricular basal diameter {RVBD}, right ventricular mid diameter {RVMD}, right ventricular longitudinal diameter {RVLD}, right ventricle outflow tract diameter (RVOTD)} and right atrial diameter (RAD), along with right atrial volumes (RAV), were measured in an apical four-chamber view as per ASE recommendations [5]. Left ventricular mass (LV mass) was calculated using the linear cube method as follows [5]: left ventricular mass in grams = 0.8 × (1.04 × {(IVSD + PWD + LVEDD)^3^ LVEDD^3^}) + 0.6. Relative wall thickness (RWT) was measured as 2 × PWD/LVEDD [15,16]. All measurements were divided by BSA to obtain indexed measurements.

Statistical analysis of data

All the parameters were recorded in the defined proforma and subjected to analysis using appropriate statistical methods. The data obtained was carefully scrutinized, categorized, coded, and statistically analyzed through the software package SPSS 19.0 version (Armonk, NY: IBM Corp.). Continuous data were expressed as mean± standard deviation. Continuous data were compared between groups using a one-way or two-way ANOVA test as appropriate. The chi-square test was used for group comparisons of discrete data. All statistical tests were two-tailed, and p < 0.05 was taken as significant.

## Results

Baseline demographic details

A total of 2245 study subjects were included in the final analysis. The mean age was 32.52±11.55 years (range = 18-60 years). There were 1100 (49%) males (mean age = 32.66±12.64 years) and 1145 (51%) females (mean age = 32.45±10.65 years). Females had smaller body surface area (1.57±0.10 vs 1.72±0.09, p = 0.00) but higher BMI (24.30±1.14 vs 23.29±0.57) compared to males. All the biochemical and hematological parameters were within the normal range and ECG was also normal.

Gender-wise comparison

Absolute Echocardiographic Parameters

Males had higher left ventricular (LV) volumes, LV mass, left atrial (LA) diameter, LA volume, RVBD, TAPSE, and aortic size, while females had higher LVEF, GLS, E, A, and e’ values. The upper normal reference limit (UNRL) of LVEDV, LVESV, SV, DT, and inferior vena cava (IVC) size was higher in males, while the UNRL of GLS, fractional shortening (FS), atrial reversal was higher in females. The UNRL of LVEDD, LVESD, IVSD, LV mass, RWT, LVEF, left ventricular outflow tract diameter (LVOTD), aorta, annulus, RVBD, RVOTD, TAPSE, LA size, E, A, e’, E/e’, right atrial (RA) size, RA volume and main pulmonary artery (MPA) was similar in both genders. The lower normal reference limit (LNRL) of LVESD, LVEDV, LVESV, LV mass, SV, LA size, and volume was higher in males, while the UNRL of FS, atrial reversal was higher in females. The UNRL of LVEDD, IVSD, PWTD, RWT, LVEF, GLS, LVOTD, aorta, annulus, RVBD, RVOTD, TAPSE, E, A, E/A, e’, E/e’, and MPA size was similar in both genders. The details are shown in Table 1.

**Table 1:** Description of various absolute echocardiographic parameters in the entire cohort and comparison by gender. LVEDD: left ventricular end-diastolic dimension; LVESD: left ventricular end-systolic dimension; LVEDV: left ventricular end-diastolic volume; LVESV: left ventricular end-systolic volume; IVSD: septal thickness at end diastole; PWTD: posterior wall thickness at end diastole; LV mass: left ventricular mass; RWT: relative wall thickness; LVEF: left ventricular ejection fraction; GLS: global longitudinal strain; FS: fractional shortening; LVOTD: left ventricular outflow tract diameter; SV: stroke volume; A-AO: ascending aorta; STJ-AO: sinotubular junction of aorta; RVBD: right ventricular basal diameter; RVMD: right ventricular mid diameter; RVL: right ventricular longitudinal diameter; RVOTD: right ventricle outflow tract diameter; RVWT: right ventricular wall free wall thickness; TAPSE: tricuspid annular plane systolic excursion; LAD: left atrial anteroposterior diameter; LAV: left atrial volume; E: mitral inflow early diastolic velocity; A: mitral inflow late diastolic velocity; MV-DT: deceleration time of mitral E velocity; e′: mitral annular early diastolic velocity; RAD: right atrial diameter; RAV: right atrial volume; IVC: inferior vena cava; MPA: main pulmonary artery

Parameters	Total (n=2245)		Male (n=1100) (49%)		Female (n=1145) (51%)		p-Value
	Range	Mean±SD	Range	Mean±SD	Range	Mean±SD	
LVEDD (mm)	37-54	44.91±3.57	37-54	44.9±3.7	37-54	44.9±3.4	0.866
LVESD (mm)	16-39	27±3.73	20-39	27.0±4.0	16-38	27.0±3.5	0.990
LVEDV (mL)	46-150	81.21±18.3	57-150	85.7±19.9	46-136	76.9±15.5	0.00
LVESV (mL)	13-52	28.32±7.11	20-52	31.0±7.1	13-40	25.8±6.1	0.00
IVSD (cm)	0.6-1.1	0.81±0.11	0.6-1.1	0.8±0.1	0.6-1.0	0.8±0.1	0.85
PWTD (cm)	0.6-1.0	0.75±0.11	0.6-1.0	0.8±0.1	0.6-0.9	0.7±0.1	0.362
LV mass (g)	56-213	125.5±32.74	63-213	129.5±34.2	56-212	121.8±31	0.029
RWT	0.23-0.47	0.33±0.05	0.2-0.5	0.33±0.0	0.2-0.5	0.33±0.0	0.198
LVEF (%)	54-82	64.67±5.24	55-82	63.4±4.7	54-80	66.1±5.4	0.00
GLS	-16 to -25	-20.01±1.82	-16 to -23	-19.7±1.8	-16 to -25	-20.3±1.8	0.00
FS (%)	22-56	39.6±5.8	22-53	39.5±5.8	25-56	39.7±5.9	0.68
LVOTD (mm)	16-25	19.75±2.09	17-25	19.7±2.4	16-25	19.8±1.8	0.818
SV (mL)	21-117	52.92±13.4	34-117	54.7±14.9	21-102	51.2±11.5	0.006
A-AO (mm)	21-33	25.63±2.75	21-33	25.9±2.8	21-32	25.4±2.6	0.032
STJ-AO (mm)	20-32	24.86±2.59	21-32	25.1±2.6	20-30	24.6±2.5	0.051
Annulus (mm)	17-25	20.31±2.38	17-25	20.5±2.4	17-25	20.2±2.3	0.188
Sinus of Valsalva (mm)	25-36	30.22±2.70	26-36	30.8±2.7	25-36	29.6±2.6	0.00
RVBD (mm)	25-35	29.85±2.64	25-35	30.4±2.6	25-35	29.3±2.5	0.00
RVMD (mm)	20-31	24.99±2.68	20-31	25.6±2.6	20-31	24.5±2.6	0.00
RVL (mm)	55-81	69.24±6.31	58-81	69.5±6.4	55-81	69.0±6.3	0.381
RVOTD (mm)	20-30	24.12±2.11	20-30	24.4±2.2	20-30	23.9±2.0	0.017
RVWT (mm)	3-5	4.01±0.81	3-5	4.1±0.8	3-5	3.9±0.8	0.081
TAPSE (mm)	17-24	19.49±1.71	17-23	19.7±1.7	17-24	19.3±1.7	0.03
LAD (mm)	23-39	31.99±3.41	26-39	33.1±3.4	23-37	31.0±3.1	0.00
LAV (mL)	24-26	35.55±4.13	29-45	36.9±3.5	24-46	34.2±4.2	0.00
E (cm/s)	64-120	83.25±12.96	64-120	79.0±10.6	64-120	87.3±13.4	0.00
A (cm/s)	50-84	64.37±7.63	50-84	61.2±5.9	50-84	67.4±7.9	0.00
E/A	1.01-1.51	1.29±0.09	1-1.5	1.3±0.1	1.1-1.5	1.3±0.1	0.631
MV-DT (ms)	120-240	166.45±27.69	120-240	169±29.8	110- 228	163.3±25	0.014
e′ (septal)	9-17	13.36±2.52	9-17	12.4±2.3	9-17	14.3±2.4	0.00
E/e′ (septal)	5-7.8	6.29±0.59	5-7.8	6.4±0.6	5-7.7	6.1±0.6	0.00
e′ (lateral)	10-23	16.88±3.73	10-23	15.8±3.5	10-23	17.9±3.7	0.00
E/e′ (lateral)	3.3-8.6	5.08±1.11	3.6-7.8	5.2±0.9	3.3-8.6	5.0±0.9	0.00
RAD (mm)	D26-43	34.04±3.6	27-43	35.1±3.9	26-42	33.0±3.0	0.00
RAV (mL)	29-46	37.55±3.97	32-46	39.1±4.1	29-45	36.1±3.2	0.00
IVC size (mm)	6-22	13.23±2.76	8-22	14.6±2.9	6-19	11.9±1.8	0.00
MPA (mm)	13-24	17.54±2.15	14-24	18.4±2.1	13-23	16.8±1.8	0.00

*Indexed Echocardiographic Parameter*s

Males had higher indexed LVESV and IVC sizes, while females had higher indexed LVEDD, LVESD, LVOTD, aorta, RVBD, LA, and RA sizes. The upper normal reference limit (UNRL) of indexed LVESV, IVC, and RA volume was higher in males, while UNRL of indexed LVEDD, LVESD, LVEDV, LV mass, LVOTD, SV, aorta, annulus, LAD, RVBD, RVOTD, RAD was higher in females; and UNRL of indexed LA volumes and MPA size was similar in both genders. The lower normal reference limit (LNRL) of indexed LVESD, LVEDV, LVESV, SV, LAV, and IVC was higher in males, while LNRL of indexed LVEDD, LVOTD, aorta, annulus, RVBD, RVOTD, RAD was higher in females; and the LNRL of indexed LV mass, MPA size was similar in both genders. The details are shown in Table 2.

**Table 2:** Description of various indexed echocardiographic parameters in the entire cohort and comparison based on gender. LVEDDI: left ventricular end-diastolic dimension indexed; LVESDI: left ventricular end-systolic dimension indexed; LVEDVI: left ventricular end-diastolic volume indexed; LVESVI: left ventricular end-systolic volume indexed; LVI mass: left ventricular indexed mass; LVOTDI: left ventricular outflow tract diameter indexed; SVI: stroke volume indexed; A-aorta I: ascending aorta indexed; STJ-AOI: sinotubular junction of aorta indexed; RVBDI: right ventricular basal diameter indexed; RVMDI: right ventricular mid diameter indexed; RVLI: right ventricular longitudinal diameter indexed; RVOTDI: right ventricle outflow tract diameter indexed; LADI: left atrial anteroposterior diameter indexed; LAVI: left atrial volume indexed; RADI: right atrial diameter indexed; RAVI: right atrial volume indexed; IVCI: inferior vena cava indexed; MPAI: main pulmonary artery indexed

Parameters	Total (n=2245)		Male (n=1100) (49%)		Female (n=1145) (51%)		p-Value
	Range	Mean±SD	Range	Mean±SD	Range	Mean±SD	
LVEDDI (mm)	20.33-36.96	27.38±2.98	20.3-33.3	26.1±2.6	21.3-37	28.6±2.8	0.00
LVESDI (mm)	9.2-24.22	16.46±2.82	12.1-22.7	15.7±2.5	9.2-24.2	17.2±2.5	0.00
LVEDVI (mL)	26.97-96.45	49.36±10.94	33.9-85.2	49.8±11.6	27-96.5	48.9±10.2	0.403
LVESVI (mL)	8.07-30.33	17.19±4.52	11.3-30.3	18.0±4.2	8-26.8	16.4±4.0	0.00
LVI mass (g)	39.56-133.33	76.11±20.02	39.6-122	75.7±20.1	39.9-133	77.5±20.2	0.152
LVOTDI (mm)	9.04-18.75	12.05±1.12	9-16.6	11.5±1.6	10.2-18.8	12.6±1.4	0.00
SVI (mL)	12.07-72.34	32.20±7.88	19.4-66.9	31.8±8.7	12-72.3	32.6±7.6	0.293
A-AOI (mm)	11.71-21.88	15.62±1.63	11.7-19.6	15.1±1.9	12.6-21.9	16.1±1.9	0.00
STJ-AOI (mm)	11.17-21.09	15.15±1.58	11.2-18.6	14.6±1.8	12-21.1	15.7±1.8	0.00
Annulus indexed (mm)	9.04-17.14	12.37±1.51	9-15.9	11.9±1.6	9.8-17.1	12.8±1.6	0.00
Sinus of Valsalva indexed	13.83-25.78	18.41±1.72	13.8-23.4	17.9±1.9	14.4-25.8	18.9±2.0	0.00
RVBDI (mm)	13.51-25	18.19±1.68	13.5-22.8	17.7±1.9	14.9-25	18.7±2.0	0.00
RVMDI mm	10.81-22.14	15.22±1.77	10.8-19.3	14.9±1.8	12.1-22.1	15.6±1.9	0.00
RVLI (mm)	31.35-60.16	42.21±3.98	31.4-54.5	40.5±4.5	32.6-60	43.9±4.7	0.00
RVOTDI (mm)	11.2-20	14.8±1.5	11.2-18.4	14.2±1.6	12.1-20	15.2±1.5	0.00
LADI (mm)	14.47-25.83	19.45±2.21	14.8-24	19.2±2.0	14.5-25.8	19.7±2.0	0.07
LAVI (mL)	16.46-28.97	21.59±2.29	17.3-28.3	21.5±2.0	16.5-29	21.7±2.5	0.258
RADI (mm)	14.84-26.58	20.68±2.3	14.8-24.8	20.4±2.1	16.4-26.6	21.0±1.9	0.004
RAVI (mL)	18.39-28.97	22.81±2.15	18.6-29	22.7±2.3	18.4-28.3	22.9±2.0	0.244
IVCI (mm)	4.8-11.89	7.97±1.52	5.1-11.9	8.4±1.4	4.8-10.6	7.5±0.8	0.00
MPAI (mm)	8.54-13.41	10.63±1.76	8.6-13.3	10.6±1.0	8.5-13.4	10.6±1.0	0.89

Echocardiographic parameters stratified into different age groups and gender

LV volumes and dimensions decreased with age in both genders. Both septal and posterior wall thicknesses and LV mass increased with age in both males and females. However, this increase was higher in females than in males. LV ejection fraction showed little variation with age. LA and RA volumes changed significantly with age in both genders. All diastolic parameters were significantly associated with age, namely, a decline in mitral peak E velocity, an increase in mitral peak A velocity, a decline in mitral E/A ratio, and an increase in mitral DT were observed with age in both males and females. TDI e’ velocities at both sides decreased significantly with age, while e’ velocity at the lateral annulus was higher than that at the septal annulus throughout all ages in both males and females. E/e’ ratio increased with age. The different echocardiographic measurements stratified by different age groups and gender are shown in Table 3.

**Table 3:** Description of various echocardiographic parameters stratified into different age groups and gender. LVEDD: left ventricular end-diastolic dimension; LVESD: left ventricular end-systolic dimension; LVEDV: left ventricular end-diastolic volume; LVESV: left ventricular end-systolic volume; IVSD: septal thickness at end-diastole; PWTD: posterior wall thickness at end-diastole; LV mass: left ventricular mass; RVBD: right ventricular basal diameter; RVMD: right ventricular mid diameter; RVOTD: right ventricle outflow tract diameter; LAD: left atrial anteroposterior diameter; LAV: left atrial volume; E: mitral inflow early diastolic velocity; A: mitral inflow late diastolic velocity; MV-DT: deceleration time of mitral E velocity; e′: mitral annular early diastolic velocity; RAD: right atrial diameter; RAV: right atrial volume

Parameters	Age (18-40 years)				Age (41-60 years)				p-Value between groups I and III	p-Value between groups II and IV
	Group I		Group II		Group III		Group IV			
	Male (n=750) (33.40%)		Female (n=905) (40.31%)		Male (n=350) (15.59%)		Female (n=240) (10.69%)			
	Mean	±SD	Mean	±SD	Mean	±SD	Mean	±SD		
LVEDD (mm)	45.20	3.47	44.39	3.67	44.20	3.25	42.00	3.26	0.001	0.0001
LVESD (mm)	27.07	3.72	26.55	3.56	26.56	3.26	25.67	3.12	0.02	0.0005
LVEDV (mL)	86.38	15.7	75.75	14.5	84.10	16.7	73.40	17.1	0.02	0.03
LVESV (mL)	31.42	7.1	25.07	7.2	30.04	7.5	23.42	8.1	0.003	0.002
IVSD (cm)	0.83	0.11	0.78	0.12	0.86	0.11	0.84	0.21	0.001	0.0001
PWTD (cm)	0.76	0.11	0.74	0.12	0.77	0.11	0.77	0.11	0.16	0.0002
LV mass (g)	129.7	31.2	116.56	32.5	130.0 0	31.2	141.46	31.1	0.88	0.0001
LVEF (%)	63.20	5.12	66.47	5.23	63.70	5.26	66.12	5.56	0.13	0.36
RVBD (mm)	30.53	2.6	29.24	2.7	30.17	2.5	29.60	2.8	0.74	0.33
RVMD (mm)	25.68	2.3	24.39	2.4	25.40	2.6	24.67	2.7	0.07	0.34
RVOTD (mm)	24.29	2.1	23.82	2.2	24.50	2.3	23.90	2.6	0.13	0.63
LAD (mm)	31.57	3.31	30.00	3.21	36.29	3.23	34.58	3.22	0.0001	0.0001
LAV (mL)	35.69	4.12	33.18	4.31	39.61	4.11	38.10	4.24	0.0001	0.0001
E (cm/s)	79.63	12.5	87.82	12.6	77.63	12.42	85.54	12.31	0.013	0.016
A (cm/s)	61.40	5.67	67.61	5.43	64.76	5.42	69.71	5.11	0.0001	0.0001
E/A	1.29	0.12	1.39	0.21	1.27	0.12	1.28	0.2	0.01	0.0001
MV-DT (ms)	168.6	25.12	162.25	24.32	172.0 6	24.11	167.21	25.12	0.0001	0.0001
e′ (septal)	12.46	2.31	14.32	2.41	12.26	2.31	14.17	2.21	0.18	0.38
E/e′ (septal)	6.46	0.51	6.17	0.42	6.56	0.41	6.27	0.42	0.0015	0.001
e′ (lateral)	17.26	3.31	18.96	3.21	12.71	3.11	15.90	3.24	0.0001	0.0001
E/e´ lateral	4.69	0.91	4.68	0.11	6.16	0.87	6.26	0.81	0.0001	0.0001
RAD (mm)	33.25	3.5	31.87	3.4	39.17	3.4	37.19	3.5	0.0001	0.0001
RAV (mL)	37.11	3.4	34.97	3.7	43.23	3.5	40.33	3.1	0.0001	0.0001

Comparison of echocardiographic parameters of study subjects with international guidelines

In Male Study Subjects

The absolute values of LV size, volumes, IVSD, PWD, and LV mass were all higher in Western studies than in our study [2,5]. Our study population had higher LVEF. Other parameters, including RV size, aortic size, LA size, and RA size, were all higher in the Western population. Among indexed parameters, LV volumes, LVESD, aortic annulus, LA, and RA volumes were still significantly higher in Western data than in the study population. The comparison of absolute echocardiographic measurements with the Western population in male study subjects is shown in Table 4. 

**Table 4:** Comparison of absolute echocardiographic parameters with the Western population in male study subjects. *ASE/EACVI [5]. **NORRE study [2]. ASE/EACVI: American Society of Echocardiography/European Association of Cardiovascular Imaging; NORRE study: Normal Reference Ranges for Echocardiography study; LVEDD: left ventricular end-diastolic dimension; LVESD: left ventricular end-systolic dimension; LVEDV: left ventricular end-diastolic volume; LVESV: left ventricular end-systolic volume; IVSD: septal thickness at end-diastole; PWTD: posterior wall thickness at end-diastole; LV mass: left ventricular mass; LVEF: left ventricular ejection fraction; A-AO: ascending aorta; STJ-AO: sinotubular junction of aorta; RVBD: right ventricular basal diameter; RVMD: right ventricular mid diameter; RVL: right ventricular longitudinal diameter; RVOTD: right ventricle outflow tract diameter; RVWT: right ventricular wall free wall thickness; TAPSE: tricuspid annular plane systolic excursion; LAD: left atrial anteroposterior diameter; LAV: left atrial volume; RAD: right atrial diameter; RAV: right atrial volume

Parameters	Study population, male (n=1100)		Studies		p-Value
	Range	Mean±SD	Range	Mean±SD	
LVEDD (mm)	37-54	44.9±3.7	42-58.4	50.2±4.1 (n=502)*	0.0001
LVESD (mm)	20-39	27.0±4.0	25-39.8	32.4±3.7 (n=389)*	0.0001
LVEDV (mL)	57-150	85.7±19.9	62-150	106±22 (n=201)*	0.0001
LVESV (mL)	20-52	31.0±7.1	21-61	41±10 (n=201)*	0.0001
IVSD (cm)	0.6-1.1	0.8±0.1	0.6-1	0.92±0.16(n=320)**	0.0001
PWTD (cm)	0.6-1.0	0.8±0.1	0.6-1	0.93±0.15(n=320)**	0.0001
LV mass (g)	63-213	129.5±34.2	88-224	145.6±36.7(n=320)**	0.0001
LVEF%	55-82	63.4±4.7	52-72	62±5 (n=201)*	0.0001
A-AO (mm)	21-33	25.9±2.8	22-36	30±4 (n=68)*	0.0001
STJ-AO (mm)	21-32	25.1±2.6	22-36	29±3 (n=68)*	0.0001
Annulus indexed (mm)	17-25	20.5±2.4	19-24	26±3 (n=68)*	0.0001
Sinus of Valsalva (mm)	26-36	30.8±2.7	29-42	34±3 (n=68)*	0.0001
RVBD (mm)	25-35	30.4±2.6	25-41	33±4 (n=695)*	0.0001
RVMD (mm)	20-31	25.6±2.6	19-35	27±4 (n=1938)*	0.0001
RVL (mm)	58-81	69.5±6.4	59-83	71±6 (n=537)*	0.0001
RVOTD (mm)	20-30	24.4±2.2	20-30	25±2.5 (n=380)*	0.0001
RVWT (mm)	3-5	4.1±0.8	1-5	3±1 (n=537)*	0.0001
TAPSE (mm)	17-23	19.7±1.7	Abnormal <17	24±3.5 (n=4803)*	0.0001
LAD (mm)	26-39	33.1±3.4	30-40	40±4.5 (n=320)**	0.0001
LAV (mL)	29-45	36.9±3.5	22-57	47.8±13(n=320)**	0.0001
RAD (mm)	27-43	35.1±3.9	29-45	38.4±5.4(n=320)**	0.0001
RAV (mm)	32-46	39.1±4.1	29-45	43.8±13.4(n=320)**	0.0001

The comparison of indexed echocardiographic parameters with the Western population in male study subjects is shown in Table 5.

**Table 5:** Comparison of indexed echocardiographic parameters with the Western population in male study subjects. *ASE/EACVI [5]. **NORRE study [2]. ASE/EACVI: American Society of Echocardiography/European Association of Cardiovascular Imaging; NORRE study: Normal Reference Ranges for Echocardiography study; LVEDDI: left ventricular end-diastolic dimension indexed; LVESDI: left ventricular end-systolic dimension indexed; LVEDVI: left ventricular end-diastolic volume indexed; LVESVI: left ventricular end-systolic volume indexed; LVI mass: left ventricular mass indexed; A-AOI: ascending aorta indexed; STJ-AOI: sinotubular junction of aorta indexed; LADI: left atrial anteroposterior diameter indexed; LAVI: left atrial volume indexed; RADI: right atrial diameter indexed; RAVI: right atrial volume indexed

Parameters	Study population, male (n=1100)		Studies		p-Value
	Range	Mean±SD	Range	Mean±SD	
LVEDDI (mm)	20.3-33.3	26.1±2.6	24-35	26±2.5 (n=320)*	0.54
LVEDSI (mm)	12.1-22.7	15.7±2.5	14-24	17±2.5 (n=320)**	0.0001
LVEDVI (mL)	33.9-85.2	49.8±11.6	34-74	54±10 (n=201)*	0.0001
LVESVI (mL)	11.3-30.3	18.0±4.2	11-31	21±5 (n=201)*	0.0001
LVI mass (g)	39.6-122	75.7±20.1	49-115	74.8±17.5 (n=320)**	0.55
A-AOI	11.7-19.6	15.1±1.9	13-17	15±2 (n=68)*	0.67
STJ-AOI (mm)	11.2-18.6	14.6±1.8	14-16	15±2 (n=68)*	0.09
Annulus indexed (mm)	9-15.9	11.9±1.6	12-14	13±1 (n=68)*	0.0001
Sinus of Valsalva indexed (mm)	13.8-23.4	17.3±1.9	17-22	17±2 (n=68)*	0.2
LADI (mm)	14.8-24	19.2±2.0	15-23	18.2±2.3 (n=320)*	0.0001
LAVI (mL)	17.3-28.3	21.5±2.0	<34	27±6 (n=320)*	0.0001
RADI (mm)	14.8-24.8	20.4±2.1	17-25	19.8±3 (n=320)*	0.0001
RAVI (mL)	18.6-29	22.7±2.3	<39	24±7 (n=320)*	0.0001

In Female Study Subjects

The absolute values of LV systolic size and volumes, PWTD, LV mass, aortic size, and aortic annulus were all higher in Western studies than in our study. Our study population had higher LVEF. Among indexed parameters, LV diastolic dimension and volumes, LV mass, Sino tubular junction, sinus of Valsalva, LA diameter, RA diameter, and volumes were significantly higher in the study subjects than in the Western data. LA volumes, however, were higher in Western subjects than in study subjects. The comparison of absolute echocardiographic parameters with the Western population in female study subjects is shown in Table 6.

**Table 6:** Comparison of absolute echocardiographic parameters with the Western population in female study subjects. *ASE/EACVI [5]. **NORRE study [2]. ASE/EACVI: American Society of Echocardiography/European Association of Cardiovascular Imaging; NORRE study: Normal Reference Ranges for Echocardiography study; LVEDD: left ventricular end-diastolic dimension; LVESD: left ventricular end-systolic dimension; LVEDV: left ventricular end-diastolic volume; LVESV: left ventricular end-systolic volume; IVSD: septal thickness at end-diastole; PWTD: posterior wall thickness at end-diastole; LV mass: left ventricular mass; LVEF: left ventricular ejection fraction; A-AO: ascending aorta; STJ-AO: sinotubular junction of aorta

Parameters	Study population, female (n=1145)		Studies		p-Value
	Range	Mean±SD	Range	Mean±SD	
LVEDD (mm)	37-54	44.9±3.4	37.8-52.2	45±3.6 (n=769)*	0.53
LVESD (mm)	16-38	27.0±3.5	21.6-34.8	28.2±3.3 (n=630)*	0.0001
LVEDV (mL)	46-136	76.9±15.5	46-106	76±15 (n=319)*	0.35
LVESV (mL)	13-40	25.8±6.1	14-42	28±7 (n=319)*	0.0001
IVSD (cm)	0.6-1.0	0.8±0.1	0.6-9	0.8±0.15 (n=414)**	>0.99
PWTD (cm)	0.6-0.9	0.7±0.1	0.6-9	0.85±0.13 (n=414)**	0.0001
LV mass (g)	56-212	121.8±31	67-162	112±30.5 (n=414)**	0.0001
LVEF (%)	54-80	66.1±5.4	54-74	64±5 (n=319)*	0.0001
A-AO (mm)	21-32	25.4±2.6	22-36	27±4 (n=67)*	0.0001
STJ-AO (mm)	20-30	24.6±2.5	22-36	26±3 (n=67)*	0.0001
Annulus indexed (mm)	17-25	20.2±2.3	17-22	23±2 (n=67)*	0.0001
Sinus of Valsalva (mm)	25-36	29.6±2.6	25-34	30±3 (n=67)*	0.22

The comparison of indexed echocardiographic parameters with the Western population in female study subjects is shown in Table 7.

**Table 7:** Comparison of indexed echocardiographic parameters with the Western population in female study subjects *ASE/EACVI [5]. **NORRE study [2]. ASE/EACVI: American Society of Echocardiography/European Association of Cardiovascular Imaging; NORRE study: Normal Reference Ranges for Echocardiography study; LVEDDI: left ventricular end-diastolic dimension indexed; LVESDI: left ventricular end-systolic dimension indexed; LVEDVI: left ventricular end-diastolic volume indexed; LVESVI: left ventricular end-systolic volume indexed; LVI mass: left ventricular mass indexed; A-AOI: ascending aorta indexed; STJ-AOI: sinotubular junction of aorta indexed; LADI: left atrial anteroposterior diameter indexed; LAVI: left atrial volume indexed; RADI: right atrial diameter indexed; RAVI: right atrial volume indexed

Parameters	Study population, female (n=1145)		Studies		p-Value
	Range	Mean±SD	Range	Mean±SD	
LVEDDI (mm)	21.3-37	28.6±2.8	22-30	26±2 (n=769)*	0.0001
LVESDI (mm)	9.2-24.2	17.2±2.5	13-21	17±2.6 (n=630)*	0.112
LVEDVI (mL)	27-96.5	48.9±10.2	29-61	45±8 (n=319)*	0.0001
LVESVI (mL)	8-26.8	16.4±4.0	8-24	16±4 (n=319)*	0.114
LVI mass (g)	39.9-133	77.5±20.2	43-95	66±16.4 (n=414)**	0.0001
A-AOI (mm)	12.6-21.9	16.1±1.9	13-19	16±3 (n=67)*	0.68
STJ-AOI (mm)	12-21.1	15.7±1.8	13-17	15±2 (n=67)*	0.002
Annulus indexed (mm)	9.8-17.1	12.8±1.6	12-14	13±1 (n=67)*	0.31
Sinus of Valsalva indexed (mm)	14.4-25.8	18.9±2.0	16-20	18±2 (n=67)*	0.004
LADI (mm)	14.5-25.8	19.7±2.0	15-23	19.2±2.4 (n=414)**	0.001
LAVI (mL)	16.5-29	21.7±2.5	16-34	25±7.2 (n=414)**	0.0001
RADI (mm)	16.4-26.6	21.0±1.9	17-25	20.2± 3 (n=414)**	0.0001
RAVI (mL)	18.4-28.3	22.9±2.0	16-33	20.2±6.7 (n=414)**	0.0001

Comparison of echocardiographic parameters with other Indian data

In Male Study Subjects

Compared to Mukherjee et al., our study population had significantly higher absolute LV dimension, LV diastolic volumes, RVOT diameter, and LA volumes while LV mass, LVEF, aortic size, and TAPSE were significantly higher in their study [8]. Compared to the multicenter INDEA study, our study population had significantly higher LV diastolic dimension, LVEDV, LVEF, and RV diameter [11]. However, they had higher TAPSE and IVSD than our study population. Compared to Sullere et al., we had lower LV sizes and volumes, LV mass, and RV size while we had higher LVEF [12]. The comparison of absolute echocardiographic parameters with other Indian data in male study subjects is shown in Table 8.

**Table 8:** Comparison of absolute echocardiographic parameters of male study subjects with other Indian data. WASE: World Alliance Societies of Echocardiography Normal Values; INDEA: Indian Normative Data of Echocardiography analyzed; LVEDD: left ventricular end-diastolic dimension; LVESD: left ventricular end-systolic dimension; LVEDV: left ventricular end-diastolic volume; LVESV: left ventricular end-systolic volume; IVSD: septal thickness at end-diastole; PWTD: posterior wall thickness at end-diastole; LV mass: left ventricular mass; LVEF: left ventricular ejection fraction; GLS: global longitudinal strain; A-AO: ascending aorta; RVOTD: right ventricle outflow tract diameter; RVBD: right ventricular basal diameter; TAPSE: tricuspid annular plane systolic excursion; LAD: left atrial anteroposterior diameter; LAV: left atrial volume; RAD: right atrial diameter; RAV: right atrial volume

Parameters	Study population, male (n=1100)		Mukherjee et al. [8] (n=773)		WASE study [17] (Indian subset; n= 115)	INDEA study [11] (n=561)	Sullere et al. [12] (n= 444)
	Range	Mean±SD	Range	Mean±SD	Range	Mean±SD	Mean±SD
LVEDD (mm)	37-54	44.9±3.7	40-48	43.6±2.0 (p=0.0001)	36-56	42.8±3.8 (p=0.0001)	47.2±4.0 (p=0.0001)
LVESD (mm)	20-39	27.0±4.0	22-34	27.7±3.1 (p=0.0001)	22-37	-	-
LVEDV (mL)	57-150	85.7±19.9	64-85	74.6±5.4 (p=0.0001)	61-165	73.8±17.8 (p=0.0001)	93.4±19.8 (p=0.0001)
LVESV (mL)	20-52	31.0±7.1	25-37	31.1±2.9 (p=0.77)	21-65	-	37.2±10.2 (p=0.0001)
IVSD (cm)	0.6-1.1	0.8±0.1	0.8-10	0.9±0.07 (p=0.0001)	0.6-1.2	0.9±0.1 (p=0.0001)	1.1±0.1 (p=0.0001)
PWTD (cm)	0.6-1.0	0.8±0.1	0.6-1.0	0.83±0.1 (p=0.001)	0.6-1.1	-	1.8±0.8 (p=0.001)
LV mass (g)	63-213	129.5±34.2	95-171	133.3±18.8 (p=0.005)	74-204	-	188.5±33.7 (p=0.005)
LVEF (%)	55-82	63.4±4.7	59-78	68.7±4.8 (p=0.0001)	57-68	61.4±5.8 (p=0.0001)	60.6±4.9 (p=0.0001)
GLS	-16 to -23	-19.7±1.8	-	-	-17 to -25	-20.7±2.9 (p=0.0001)	-
A-AO (mm)	21-33	25.9±2.8	23-33	27.9±2.5 (p=0.0001)	-	-	-
RVOTD (mm)	20-30	24.4±2.2	11-23	17.4±3.0 (p=0.0001)	-	-	28.1±2.8 (p=0.0001)
RVBD (mm)	25-35	30.4±2.6	-	-	-	26.3±4.6 (p=0.0001)	-
TAPSE (mm)	17-23	19.7±1.7	Abnormal <17	24±3.5 (p=0.0001)	-	21.5±2.3 (p=0.0001)	-
LAD (mm)	26-39	33.1±3.4	17-32	24.5±3.6 (p=0.0001)	-	-	33.1±3.2 (p=0594)
LAV (mL)	29-45	36.9±3.5	11-43	26.9±7.9 (p=0.0001)	-	-	34.1±7.8 (p=0.0001)
RAD (mm)	27-43	35.1±3.9	29-45	-	-	-	-
RAV (mm)	32-46	39.1±4.1	29-45	-	-	-	-

Comparing indexed measurements with Mukherjee et al., we found that they had higher LV diastolic and systolic dimensions, volumes, and LV mass while our study subjects had higher RV size, LA dimension, and volumes [8]. Compared to the INDEA study, our study participants had higher LV dimensions, LV volumes, RV diameters, and LA volumes [11]. Compared to Sullere et al., we had lower LV systolic size and LV mass, while we had higher LA volume [12]. The comparison of indexed echocardiographic parameters with other Indian data in male study subjects is shown in Table 9.

**Table 9:** Comparison of indexed echocardiographic parameters of male study subjects with other Indian data. WASE: World Alliance Societies of Echocardiography Normal Values; INDEA: Indian Normative Data of Echocardiography Analyzed; LVEDDI: left ventricular end-diastolic dimension indexed; LVESDI: left ventricular end-systolic dimension indexed; LVEDVI: left ventricular end-diastolic volume indexed; LVESVI: left ventricular end-systolic volume indexed; LVI mass: left ventricular mass indexed; A-AOI: ascending aorta indexed; LADI: left atrial anteroposterior diameter indexed; LAVI: left atrial volume indexed

Parameters	Study population, male (n=1100)		Mukherjee et al. [8] (n=773)		WASE study [17] (n=115)	INDEA study [11] (n=561)	Sullere et al. [12] (n=444)
	Range	Mean±SD	Range	Mean±SD	Range	Mean±SD	Mean±SD
LVEDDI (mm)	20.3-33.3	26.1±2.6	24-41	32.1±4.3 (p=0.0001)	18-31	24.9±2.9 (p=0.0001)	-
LVESDI (mm)	12.1-22.7	15.7±2.5	16-27	20.4±3.5 (p=0.0001)	12-20	-	-
LVEDVI (mL)	33.9-85.2	49.8±11.6	43-67	54.9±6.0 (p=0.0001)	29-62	42.8±10.7 (p=0.0001)	49.0±11.5 (p=0.21)
LVESVI (mL)	11.3-30.3	18.0±4.2	16-36	22.9±3.4 (p=0.0001)	10-24	-	19.5±5.7 (p=0.0001)
LVI (mass)	39.6-122	75.7±20.1	61-37	98.1±19.3 (p=0.0001)	40-88	-	99.1±20.7 (p=0.0001)
A-AO (mm)	11.7-19.6	15.1±1.9	14-27	-	-	-	-
RVBDI (mm)	13.5-22.8	17.7±1.9	-	-	-	15.5±2.8 (p=0.0001)	-
RVOTDI (mm)	11.2-18.4	14.2±1.6	7-18	12.8±2.7 (p=0.0001)	-	-	-
LADI (mm)	14.8-24	19.2±2.0	11-26	18.1±3.7 (p=0.0001)	-	-	-
LAVI (mL)	17.3-28.3	21.5±2.0	4-36	19.9±8.2 (p=0.0001)	-	17.8±4.2 (p=0.0001)	17.8±4.4

In Female Study Subjects

Compared to Mukherjee et al., our study subjects had significantly higher absolute LV dimension, LV diastolic volumes, RVOT diameter, and LA volumes, while LV mass, LVEF, and aortic size were significantly higher in their study population [8]. Compared to the multicenter INDEA study, our study population had significantly higher LV diastolic dimension, LVEDV, LVEF, and RV diameter. However, they had higher IVSD and GLS than our study population [11]. Compared to Sullere et al., we had lower LV diastolic sizes and volumes, LV mass, and RV size while we had higher LVEF and LA volume [12]. The comparison of absolute echocardiographic parameters with other Indian data in female study subjects is shown in Table 10.

**Table 10:** Comparison of absolute echocardiographic parameters of female study subjects with other Indian data. WASE: World Alliance Societies of Echocardiography Normal Values; INDEA: Indian Normative Data of Echocardiography analyzed; LVEDD: left ventricular end-diastolic dimension; LVESD: left ventricular end-systolic dimension; LVEDV: left ventricular end-diastolic volume; LVESV: left ventricular end-systolic volume; IVSD: septal thickness at end-diastole; PWTD: posterior wall thickness at end-diastole; LV mass: left ventricular mass; LVEF: left ventricular ejection fraction; GLS: global longitudinal strain; A-AO: ascending aorta; RVOTD: right ventricle outflow tract diameter; RVBD: right ventricular basal diameter; LAD: left atrial anteroposterior diameter; LAV: left atrial volume

Parameters	Study population, female (n=1145)		Mukherjee et al. [8] (n=604)		WASE study [17]	INDEA study [11] (n=319)	Sullere et al. [12] (n=263)
	Range	Mean±SD	Range	Mean±SD	Range	Mean±SD	Mean±SD
LVEDD (mm)	37-54	44.9±3.4	40-47	43.6±1.9 (p=0.0001)	32-49	41.2±3.9 (p=0.0001)	44.4±4.3 (p=0.03)
LVESD (mm)	16-38	27.0±3.5	22-34	27.7±3.1 (p=0.0001)	20-32	-	-
LVEDV (ml)	46-136	76.9±15.5	65-84	74.5±4.9 (p=0.0001)	40-91	67.4±16.3 (p=0.0001)	79.0±19.1 (p=0.0001)
LVESV (ml)	13-40	25.8±6.1	23-33	28.0±2.5 (p=0.0001)	14-35	-	30.4±9.6 (p=0.0001)
IVSD (cm)	0.6-1.0	0.80±0.1	0.8-1	0.9.0±0.07 (p=0.0001)	0.5-1	0.88±0.11 (p=0.0001)	1.02±0.12 (p=0.0001)
PWTD (cm)	0.6-0.9	0.7±0.1	0.6-1	0.8.2±0.1 (p=0.0001)	0.5-1	-	1.02±0.1 (p=0.0001)
LV mass (g)	56-212	121.8±31	95-172	133.4±19.1 (p=0.0001)	48- 125	-	157.2±36.0 (p=0.0001)
LVEF (%)	54-80	66.1±5.4	58-80	69.1±5.5 (p=0.0001)	58-68	62.3±6.1 (p=0.0001)	62.2±5.1 (p=0.0001)
GLS	-16 to -25	-20.3±1.8	-	-	-	-21.5±2.8 (p=0.0001)	-
A-AO (mm)	21-32	25.4±2.6	23-33	28.1±2.5 (p=0.0001)	-	-	-
RVOTD (mm)	20-30	23.9±2.0	13-23	17.4±2.7 (p=0.0001)	-	-	25.9±3.2 (p=0.0001)
RVBD (mm)	25-35	29.3±2.5	-	-	-	25.3±4.3 (p=0.0001)	-
LAD (mm)	23-37	31.0±3.1	17-32	24.5±3.7 (p=0.0001)	-	-	30.8±3.6 (p=0.0001)
LAV (ml)	24-46	34.2±4.2	11-43	26.9±8.1 (p=0.0001)	-	-	31.7±7.8 (p=0.0001)

Comparing indexed measurements with Mukherjee et al., we found that they had higher LV diastolic and systolic dimensions, volumes, and LV mass while our study subjects had higher RV size, LA dimension, and volumes [8]. Compared to the INDEA study, our study participants had higher LV dimensions, LV volumes, RV diameters, and LA volumes [11]. Compared to Sullere et al., we had lower LV systolic size and LV mass, while we had higher LA volume [12]. The comparison of indexed echocardiographic parameters with other Indian data in female study subjects is shown in Table 11.

**Table 11:** Comparison of indexed echocardiographic parameters of female study subjects with other Indian data. WASE: World Alliance Societies of Echocardiography Normal Values; INDEA: Indian Normative Data of Echocardiography analyzed; LVEDDI: left ventricular end-diastolic dimension indexed; LVESDI: left ventricular end-systolic dimension indexed; LVEDVI: left ventricular end-diastolic volume indexed; LVESVI: left ventricular end-systolic volume indexed; LVI mass: left ventricular mass indexed; A-AOI: ascending aorta indexed; RVOTDI: right ventricular outflow tract diameter indexed; RVBDI: right ventricular basal diameter indexed; LADI: left atrial anteroposterior diameter indexed; LAVI: left atrial volume indexed

Parameters	Study population, female (n=1145)		Mukherjee et al. [8] (n=604)		WASE study [17]	INDEA study [11] (n=319)	Sullere et al. [12] (n=263)
	Range	Mean±SD	Range	Mean±SD	Range	Mean±SD	Mean±SD
LVEDDI (mm)	21.3-37	28.6±2.8	24-40	32.1±3.9 (p=0.0001)	20-32	25.8±2.8 (p=0.0001)	-
LVESDI (mm)	9.2-24.2	17.2±2.5	14-27	20.4±3.1 (p=0.0001)	12-21	-	-
LVEDVI (mL)	27-96.5	48.9±10.2	43-66	54.8±5.6 (p=0.0001)	26-58	42.0±10.1 (p=0.0001)	46.8±12.4 (p=0.0001)
LVESVI (mL)	8-26.8	16.4±4.0	14-27	20.6±3.1 (p=0.0001)	9-22	-	18.1±6.0 (p=0.0001)
LVI mass (g)	39.9-133	77.5±20.2	62-135	98.3±18.3 (p=0.0001)	37-78	-	93.3±24.2 (p=0.0001)
A-AOI (mm)	12.6-21.9	16.1±1.9	15-27	20.7±3.0 (p=0.0001)	-	-	-
RVOTDI (mm)	12.1-20	15.2±1.5	8-18	12.9±2.5 (p=0.0001)	-	-	-
RVBDI (mm)	14.9-25	18.7±2.0	-	-	-	15.6±3.0 (p=0.0001)	-
LADI (mm)	14.5-25.8	19.7±2.0	11-25	18.0±3.3 (p=0.0001)	-	-	-
LAVI (mL)	16.5-29	21.7±2.5	3-37	19.8±8.5 (p=0.0001)	-	18.6±4.6 (p=0.0001)	18.7±5.0 (p=0.0001)

## Discussion

Our study is the first to comprehensively analyze cardiac chamber quantification in a large cohort of healthy volunteers with representation from both genders and over a wide range of ages in the Kashmiri population. Our observations enforce the need to develop ethnic-specific reference values for different populations.

Comparison by age and gender in the study population

Left Ventricular (LV) Size

Males had higher absolute LV volumes and LV mass. However, when indexed to body surface area (BSA), only LVESV remained higher in males, while other parameters became higher in females. This is in contrast to other Western [5] and Indian studies [9,16,17], which have shown that LV volumes and dimensions remained higher despite indexing to BSA; however, the study by Mukherjee et al. also did not show significant gender difference when indexed to BSA [8]. Females were noted to have higher LVEF and GLS than males. LV mass increased with age in both males and females. However, this increase was higher in females than males [18]. Overall, the absolute upper and lower reference limits were higher in males than females with age-related changes, highlighting the importance of applying age-gender-specific reference values for reliable identification of cardiac chamber enlargement and dysfunction. The indexed upper and lower cut-off values were different in males than females, suggesting that patients with valvular heart disease indexing for body surface area alone might be insufficient to identify LV impairment.

Right Ventricular (RV) Size

Although the absolute RV size and RV wall-free thickness were higher in males, the indexed sizes were higher in females. This was due to the lower BSA of females. There was no significant difference in RV parameters with different age groups. This is in contrast to different studies [18-20]. However, the INDEA study also reported findings consistent with ours [11]. Previous studies have also shown larger absolute RV sizes in males than females, with minimal differences when indexed to BSA between the two genders. It may be worth mentioning that ASE/EACVI do not recommend the use of indexing to BSA for RV dimensions. TAPSE was higher in males as compared to females.

Left Atrial (LA) Size and Left Ventricular Diastolic Function

The absolute values of LA size and volumes were higher in males than females. However, after indexing, there was no significant difference, as demonstrated in other studies [11,21-23]. We also noted that both LA size and LA volume significantly increased in males and females with age, which was in contrast with other studies [18]. Our data suggests that there is a need for checking age-specific references in our population. Females had higher E, A, and e’ velocities and shorter deceleration time. However, E/A ratio was similar in both genders. Similar findings were noted in the INDEA study [11]. All diastolic parameters were significantly associated with age. TDI e’ velocities at both sides decreased significantly with age. The e’ velocity at the lateral annulus was higher than that at the septal annulus throughout all ages in both males and females. Increased LA size and volume and change in diastolic parameters with age may be due to altered atrial and diastolic mechanics with age [24].

Right Atrial (RA) Size

The absolute values of RA size and volume were higher in males than females. However, after indexing, there was no significant difference. There was also a significant correlation of age with both RA size and volumes in both genders, thus suggesting the need to check age-specific references in assessing RA size in our population.

Comparison with Western studies

In Males

We noted significantly lower absolute values of LV, RV, LA, RA, and aortic size in study subjects compared to the Western population [5,18], which confirms the need for separate echocardiographic reference ranges for our population. Even after indexing to BSA, LV volumes, LVESD, LA, and RA volumes, aortic annulus remained significantly higher in the western population [5,18].

In Females

Although the absolute values of LV systolic size and volumes, PWD, LV mass, aortic size, and aortic annulus were all higher in Western studies than our study, the indexed parameters, including LV diastolic dimension and volumes, LV mass, Sino tubular junction, sinus of Valsalva, LA diameter, RA diameter and volumes were still significantly higher in study subjects than Western data [5,18]. This is due to lower BSA of our female study subjects as compared with the West. Our population also had significantly higher LVEF.

Implications of Disparities Between Study Subjects and Western Data

Our observations highlight and reinforce the need for developing reference values specifically for our local population and other Indian populations. For instance, the lower indexed LV values have major impact on our valvular heart disease patients. Following Western guidelines only may result in delay in surgical management of these patients. So, we propose the development of ethnic-specific guidelines for the management of valvular heart disease patients.

Comparison with other Indian studies

In Males

Our study subjects had significantly higher absolute values for LV dimension, LV diastolic volumes, RVOT diameter, and LA volumes and significantly lower LVEF and TAPSE when we compared with both Mukherjee et al.'s study [8] and multicenter INDEA study [11]. However, the population studied by Sullere, et al. from central India had significantly higher LV sizes and volumes, LV mass, and RV size than our study subjects [12].

In Females

Our study subjects had significantly higher absolute values for LV dimension, LV diastolic volumes, RVOT diameter, and LA volumes when we compared with Mukherjee et al.'s study [8] and the multicenter INDEA study [11]. However, the population studied by Sullere et al. from central India had significantly higher LV sizes and volumes, LV mass, and RV size than our study subjects [12]. Comparing indexed parameters, we had higher RV size and LA volumes and lower LV diastolic and systolic dimensions and volumes and LV mass compared to Mukherjee et al. [8].

Implications of Disparities Between Study Subjects and Other Indian Data

We found significant heterogeneity in different echocardiographic parameters when we compared our study subjects (Northern India) with Mukherjee et al. (Eastern India) [8] and Sullere et al. (Central Indian population {Madhya Pradesh}) [12] and the multicenter INDEA study [11]. While it is difficult to summarize the differences, in general, our study subjects from north India had higher absolute values for LV size, RVOTD, and LA volumes than the Eastern India population and multicenter INDEA study subjects; however, the central Indian population studied by Sullere et al. had higher left and right ventricle size than rest of Indian population [12]. Our findings have significant implications. India has a huge burden of rheumatic valvular heart disease. Our observations highlight the need to develop different reference values for different populations.

Limitations

This study was a single-center study. However, our hospital is the largest volume cardiac center in the state, and our sample size was larger than other Indian studies. There was an omission of three-dimensional echocardiography measurements from our study. Age matching was not done while comparing our data with other international and national data. Besides, the intra-observer and inter-observer variability for comparison with other studies remains a problem.

## Conclusions

The present study found significant age and gender differences in various echocardiographic measurements in our study population. There were significant differences between Western as well as other Indian populations. We suggest the development of different reference values for diverse ethnic populations. Besides, we provide normal reference values for our local population, which can be used for future reference.

## References

[1] Vasan RS, Levy D, Larson MG, Benjamin EJ (2000). Interpretation of echocardiographic measurements: a call for standardization. Am Heart J.

[2] Lancellotti P, Badano LP, Lang RM (2013). Normal Reference Ranges for Echocardiography: rationale, study design, and methodology (NORRE study). Eur Heart J Cardiovasc Imaging.

[3] Okrah K, Vaughan-Sarrazin M, Cram P (2010). Trends in echocardiography utilization in the Veterans Administration Healthcare System. Am Heart J.

[4] Lancellotti P (2014). Normal reference ranges for echocardiography: do we really need more?. Eur Heart J Cardiovasc Imaging.

[5] Lang RM, Badano LP, Mor-Avi V (2015). Recommendations for cardiac chamber quantification by echocardiography in adults: an update from the American Society of Echocardiography and the European Association of Cardiovascular Imaging. J Am Soc Echocardiogr.

[6] Daimon M, Watanabe H, Abe Y (2008). Normal values of echocardiographic parameters in relation to age in a healthy Japanese population: the JAMP study. Circ J.

[7] Ilercil A, O'Grady MJ, Roman MJ (2001). Reference values for echocardiographic measurements in urban and rural populations of differing ethnicity: the Strong Heart Study. J Am Soc Echocardiogr.

[8] Mukherjee A, Halder SK, Nandi S, Mandal M, Khanra D, Biswas K (2021). A study on normal reference values of echocardiographic chamber dimensions in young eastern Indian adults. Indian Heart J.

[9] Chahal NS, Lim TK, Jain P, Chambers JC, Kooner JS, Senior R (2012). Population-based reference values for 3D echocardiographic LV volumes and ejection fraction. JACC Cardiovasc Imaging.

[10] Bansal M, Mohan JC, Sengupta SP (2016). Normal echocardiographic measurements in Indian adults: how different are we from the western populations? A pilot study. Indian Heart J.

[11] Sengupta SP, Burkule N, Bansal M (2021). Normative values of cardiac chamber dimensions and global longitudinal strain in Indians: the Indian Normative Data of Echocardiography Analyzed (INDEA) study. Int J Cardiovasc Imaging.

[12] Sullere V, Jain D, Sullere S, Anthony C (2018). Global longitudinal strain, ejection fraction, effort tolerance and normal echocardiography measurements in healthy Indians. Indian Heart J.

[13] Rudski LG, Lai WW, Afilalo J (2010). Guidelines for the echocardiographic assessment of the right heart in adults: a report from the American Society of Echocardiography endorsed by the European Association of Echocardiography, a registered branch of the European Society of Cardiology, and the Canadian Society of Echocardiography. J Am Soc Echocardiogr.

[14] Burkule N, Bansal M, Mehrotra R, Venkateshvaran A (2017). Indian academy of echocardiography performance standards and recommendations for a comprehensive transthoracic echocardiographic study in adults. J Indian Acad Echocardiogr Cardiovasc Imaging.

[15] Devereux RB, Alonso DR, Lutas EM, Gottlieb GJ, Campo E, Sachs I, Reichek N (1986). Echocardiographic assessment of left ventricular hypertrophy: comparison to necropsy findings. Am J Cardiol.

[16] Chahal NS, Lim TK, Jain P, Chambers JC, Kooner JS, Senior R (2010). Ethnicity-related differences in left ventricular function, structure and geometry: a population study of UK Indian Asian and European white subjects. Heart.

[17] Asch FM, Miyoshi T, Addetia K (2019). Similarities and differences in left ventricular size and function among races and nationalities: results of the World Alliance Societies of Echocardiography Normal Values Study. J Am Soc Echocardiogr.

[18] Kou S, Caballero L, Dulgheru R (2014). Echocardiographic reference ranges for normal cardiac chamber size: results from the NORRE study. Eur Heart J Cardiovasc Imaging.

[19] Kawut SM, Lima JA, Barr RG (2011). Sex and race differences in right ventricular structure and function: the multi-ethnic study of atherosclerosis-right ventricle study. Circulation.

[20] Maffessanti F, Muraru D, Esposito R (2013). Age-, body size-, and sex-specific reference values for right ventricular volumes and ejection fraction by three-dimensional echocardiography: a multicenter echocardiographic study in 507 healthy volunteers. Circ Cardiovasc Imaging.

[21] D'Andrea A, Riegler L, Rucco MA (2013). Left atrial volume index in healthy subjects: clinical and echocardiographic correlates. Echocardiography.

[22] Aurigemma GP, Gottdiener JS, Arnold AM, Chinali M, Hill JC, Kitzman D (2009). Left atrial volume and geometry in healthy aging: the Cardiovascular Health Study. Circ Cardiovasc Imaging.

[23] Aune E, Baekkevar M, Roislien J, Rodevand O, Otterstad JE (2009). Normal reference ranges for left and right atrial volume indexes and ejection fractions obtained with real-time three-dimensional echocardiography. Eur J Echocardiogr.

[24] Nagueh SF (2018). Classifcation of left ventricular diastolic dysfunction and heart failure diagnosis and prognosis. J Am Soc Echocardiogr.

